# PROTOCOL: Language interventions for improving oral language outcomes in children with neurodevelopmental disorders: A systematic review

**DOI:** 10.1002/cl2.1062

**Published:** 2019-11-11

**Authors:** Anders Nordahl‐Hansen, Enrica Donolato, Arne Lervåg, Courtenay Frazier Norbury, Monica Melby‐Lervåg

**Affiliations:** ^1^ Faculty of Education Østfold University College Halden Østfold Norway; ^2^ Department of Special Needs Education University of Oslo Oslo Norway; ^3^ Institute of Education University of Oslo Oslo Norway; ^4^ Chandler House University College London London UK

## 1 BACKGROUND

### 1.1 The problem, condition or issue

This protocol presents the plan for a systematic review that will investigate the effect of oral language interventions for children with intellectual disability (ID), language disorder (LD), autism spectrum disorder (ASD), Down syndrome (DS), Williams syndrome (WS), and fragile X syndrome (FXS). Language development is a highly frequent area of difficulty for children within these diagnostic groups, and oral language interventions are therefore important. However, to provide better evidence‐informed practice, we need to investigate what oral language interventions are effective and for whom. The systematic review will not only investigate the effect of oral language interventions targeted at specific disorders but also identify interventions that may be yield similar improvements in different neurodevelopmental disorders.

Language is a crucial skill to master in childhood. Many studies emphasise that language content, structure and functional use (pragmatics) in communication is important as a foundation for other key cognitive and social achievements (Stothard, Snowling, Bishop, Chipchase, & Kaplan, 1998). Language is obviously important for the child to communicate needs, participate in social interaction, engage in play, and share information and opinions with others (Bruner, 1975). In addition, language is a crucial pathway for developing other skills such as reading comprehension (Duff, Reen, Plunkett, & Nation, 2015; Lepola, Lynch, Kiuru, Laakkonen, & Niemi, 2016; Nation & Norbury, 2005). Further, as noted by Hulme and Snowling (2013), a child with a poor oral language will not acquire reading skills nor be able to fully participate socially.

Language deficits are quite common and thus frequently encountered at community child development clinics (O'Hare, 2013). Black, Vahratian, and Hoffman (2015) reported on data from the National Health Interview Survey in the US finding that 3.3% reported their child between 3–17 years old, to have experienced language problems during the past year. A recent population‐based survey conducted in England estimated the prevalence of children having language problems of a currently unknown cause to be 7.58% (consistent with previous epidemiological studies of “specific language impairment” conducted in North America (Beitchman, Nair, Clegg, & Patel, 1986; Tomblin et al., 1997), whereas 2.34% had language deficits as part of another condition (Norbury et al., 2016). The latter group had more severe language deficits and were more likely to have co‐occurring nonverbal IQ deficits and social, emotional and behavioural problems. They were also more likely to be receiving special education support, though not necessarily more specific speech‐language therapy. Another study by Norbury et al. (2015) showed that teacher‐rated language problems was the single best predictor of academic success during the first year of school. A large portion of these children belong under the umbrella terms of developmental disorders or neurodevelopmental disorders (Bishop & Rutter, 2008; D'Souza & Karmiloff‐Smith, 2017). Some of these diagnoses have a known genetic or acquired aetiology, such as DS and FXS, whereas other diagnoses, such as LD, ID, ASD, and WS, have less understood, multifactorial aetiologies (Thapar & Rutter, 2015). However, one commonality among these groups is that they often display language difficulties and are thus in need of systematic support and interventions that target oral language.

### 1.2 Oral Language

In the present review, we focus on how oral language interventions may change oral language skills in different neurodevelopmental disorders in which children frequently present with clinically and educationally significant language difficulties. Oral language is a multi‐faceted system that comprises vocabulary (semantics), grammar (syntax and morphology) and discourse processing (pragmatics), in both expressive (language production) and receptive (language comprehension) domains. In the course of language development receptive and expressive language go hand in hand, although comprehension of language starts to develop slightly earlier than expressive skills (Hulme & Snowling, 2013). The development of vocabulary is a core ingredient in language development (Marchman & Fernald, 2008; Melby‐Lervåg & Lervåg, 2014), and measures of expressive and receptive vocabulary are widely used in intervention research that targets children with neurodevelopmental disorders. In addition to vocabulary development, oral language skills encompass grammar, which includes morphological (word formation) and syntactic (sentence formation) development (Hulme & Snowling, 2014; Scarborough, Fletcher‐Campbell, Soler, & Reid, 2009) and pragmatics. Pragmatics refers to use of language in context. While it usually assumes a social purpose, some aspects of pragmatics can rely more on oral language skills, for example, inferencing, lexical ambiguity resolution from contextual cues, or understanding non‐literal language (idioms and metaphors) (Matthews, Biney, & Abbot‐Smith, 2018).

As such, the development of oral language involves a complex process of acquiring receptive and expressive vocabulary and interpretation of lexical information in context, as well as grammar and discourse. Due to the breadth of what lies within language in this broadly defined manner, we planned for an extensive inclusion of outcome variables

### 1.3 Typical and atypical development of language

The acquisition of language is a complex but robust process that, for typically developing children, transitions smoothly over the course of development (Hulme & Snowling, 2013).

Speech perception and the making of sounds such as babbling during the first year of life are shaped and eventually turn into first words around a child's first birthday. Furthermore, a vocabulary spurt has been considered common during the second year of life (Goldfield & Reznick, 1990). However, despite commonalities in typically developing children's language acquisition, there may also be large variation (Nelson, 1981). For instance, first words may occur early for some individuals and later for others (Fenson et al., 1994), although later first words are not necessarily a call for concern. Many children show gradual development of word learning without clear spurts of vocabulary (Ganger & Brent, 2004), and multi‐word utterances have a broad age span of onset (18–24 months; Norbury & Paul, 2015). Nevertheless, language development does follow highly similar developmental patterns in typically developing children (Nelson, 1981). Studies of unselected samples typically show pervasive stability in the development of language with an almost unchanged rank order among children from the age of 4 onwards (Bornstein, Hahn, & Haynes, 2004). Before the age of 4, the developmental trajectories tend to be less stable (Duff et al., 2015). There are also studies that show that many children with a language delay at the age of 4–5.5 later resolve these issues without intervention (Bishop & Edmundson, 1987). Importantly, the language trajectories for children with neurodevelopmental disorders are complex, and there are small to substantial differences in language acquisition both within and across disorders. Additionally, many studies show that there can be pervasive deficits within different subcomponents of language for these children, necessitating assessment across the subcomponents of oral language (Norbury & Paul, 2015). However, assessing language skills in young children in a reliable and valid way is complex.

### 1.4 The value of cross‐disordered samples

In this systematic review, we will compare oral language interventions for children with different neurodevelopmental disorders. The recent CATALISE consortium work aimed at achieving consensus in diagnostic criteria and terminology for LDs (Bishop, Snowling, Thompson, Greenhalgh, & the CATALISE consortium, 2016; Bishop, Snowling, Thompson, Greenhalgh, & the CATALISE‐2 consortium, 2017) highlighted assumptions that children with different neurodevelopmental disorders require different therapeutic approaches, or that children with nonverbal cognitive deficits do not benefit from oral language interventions to the same extent that cognitively able peers do. However, there is currently limited evidence directly comparing intervention effects across neurodevelopmental disorders on which to make this judgement.

The focus on cross‐disordered samples has its value as comparison of children from different neurodevelopmental disorders enables investigation of unique approaches versus similar approaches. Several primary studies of language profiles have included direct comparison of different neurodevelopmental disorders. For instance, one study compared children with WS and children with specific language impairments and reported distinct patterns of syntactic binding (Ring & Clahsen, 2005). Differences in language have also been reported between children with FXS and DS, and autism symptom severity was associated with language differences between groups (Martin, Losh, Estigarribi, Sideris, & Roberts, 2013; Price, Roberts, Vandergrift, & Martin, 2007). On the other hand, children with ASD, DS, WS, FXS, or an ID all display some degree of language deficit (Abbeduto, McDuffie, Thurman, & Kover, 2016, 2006; Rice, Warren, & Betz, 2005). Another reason to focus on children with different neurodevelopmental disorders is that there is considerable overlap in symptomatology (Gibson, Adams, Lockton, & Green, 2013), shared aetiological risk factors (Valenti, de Bari, De Filippis, Henrion‐Caude & Vacca, 2014) and commonalities in cognitive development (Raitano Lee, Maiman, & Godfrey, 2016). Additionally, there are high rates of comorbidity amongst these groups of children (Abbeduto et al., 2016; American Psychiatric Association, 2013), and the different diagnoses are not as distinct as once thought (Thapar & Rutter, 2015). Nevertheless, whether similar oral language interventions provide similar levels of benefit for children with different neurodevelopmental disorders or whether different interventions are needed remains an unanswered question (Bishop et al., 2017).

### 1.5 The systematic review includes the following neurodevelopmental disorders

#### 1.5.1 Multi‐factorial disorders without known genetic aetiology

*LD* is the diagnostic term used in the DSM‐5 for children that show deficits in receptive or expressive language in vocabulary, sentence structure, or discourse (APA, 2013). Depending on diagnostic criteria and cut‐offs, prevalence rates vary greatly with reports ranging from 2% (Weindrich, Jennen‐Steimetz, Laucht, Esser, & Schmidt, 2000) to 31% (Jessup, Ward, Cahill, & Keating, 2008). Following new DSM‐5 criteria, a recent population study estimated the prevalence of children having a developmental LD of unknown origin to be approximately 7.58%, with an additional 2.34% occurring in the context of an existing medical diagnosis (Norbury et al., 2016).

However, the debate surrounding diagnostic criteria and terminology is ongoing (Bishop, Snowling, Thompson, Greenlagh, & Catalise consortium, 2016). Although in this review we use the DSM‐5 terminology of LD, it is important to note that we also take into account studies of children where other labels are used, such as developmental LD, receptive LD, and specific language impairments to name a few (see Bishop, 2014 for discussion and variations of terms).

The criteria for LD include problems in spoken and written communication starting early on in the developmental period. Further, the difficulties cannot be explained by sensory impairments such as hearing loss, motoric dysfunction, or another medical or neurological condition (APA, 2013). The core criteria relate to limited expressive or receptive oral language (vocabulary, grammar, and discourse) and as noted by Norbury and Paul (2015), these children are typically slow to acquire first words and first word combinations. During the course of development into the school years, vocabulary remains limited and is accompanied by varying degrees of grammatical error, error and immaturity in production, poor narrative and discourse understanding and production, and limitations in pragmatics, especially when linguistic context is important for processing (e.g., inferencig) (APA, 2013).

*ID* or Intellectual Developmental Disorder (IDD) as it will be named in the forthcoming ICD‐11 has replaced the term mental retardation. ID is a heterogeneous condition with multiple possible causes that affect cognitive functioning. Prevalence estimates in the overall population is reported to be approximately 1–3% of the population (Moeschler & Shevell, 2014). Variations in prevalence are due to differences in how the term ID is defined. In the present review, we define ID as comprising ID, global developmental delay (GDD; typically reserved for children under 5 years of age due to difficulties in reliable assessment) and unspecified ID (IDD; mainly reserved for children above 5 years of age). These disorders all reside within the collective term of ID in DSM‐5 (APA, 2013).

The defining features of ID in the DSM‐5 are (a) deficits in intellectual functions such as reasoning, learning, and abstract thinking, (b) deficits in adaptive functioning, and (c) that these deficits occur during the developmental period (APA, 2013). ID is further defined through the use of specifiers on the basis of each individual's adaptive functioning. The specifiers indicate the severity level ranging from mild to moderate to severe to profound (APA, 2013). Individuals may change in severity level status, but ID is thought to be a lifelong condition. However, interventions for children with ID can alter developmental outcomes (Eldevik, Jahr, Eikeseth, Hastings, & Hughes, 2010).

Notably, some studies use other terms such as general learning disorders, severe learning disorders and other related labels. We will include these studies if the studies describe participants in a way that fits with ICD and DSM criteria for IDD and ID.

*ASD i*s an umbrella term that has been used for some time but reached a more formal definition in 2013 following the publication of the DSM‐5 (APA, 2013). The broad spectrum encompasses disorders previously labelled as childhood autism/autistic disorder, high‐functioning autism, atypical autism, Asperger's disorder and pervasive neurodevelopmental disorder not otherwise specified.

Some epidemiological studies report a worldwide prevalence of approximately 50 to 70 per 10,000 (Elsabbagh et al., 2012; Fombonne, Quirke, & Hagen, 2009) for the broader definition of the autism spectrum. In some parts of the UK and the US, the prevalence has been reported to be more than 100 per 10,000 (Baird et al., 2006; Kogan et al., 2009) and as high as 157 per 10,000 children when statistically controlling for unknown cases (Baron‐Cohen et al., 2009; Fombonne, 2009). Thus, ASD is today regarded as one of the most common neurodevelopmental disorders (Lord & Bishop, 2010).

Two areas of functioning and behaviours make up the core diagnostic criteria of ASD. One area is made up of restricted, repetitive behaviours and interests. The second core criterion for ASD relates to social communication and social interaction (APA, 2013).

Language is an important component in the disorder and intertwined with the difficulties these children face in the social communicative domain. In the 1990s, reports indicated that approximately 50% of children with autism did not acquire functional speech (Prizant, 1996; Rapin, 1991). Today, the number of children not acquiring functional speech is lower, but still estimated to be approximately 30% (Pickles, Anderson, & Lord, 2014). This change may be due to earlier detection and intervention, but also due to broader diagnostic criteria. Even when children with ASD acquire spoken language, many have language deficits that are similar to those seen in LD. For example, Loucas et al. (2008) reported that in a sample of children with ASD with IQ scores above 80, 41 children had language impairments whereas 31 children did not. Thus, some type of language difficulty is common for children with ASD (Luyster, Kadlec, Carter, & Tager‐Flusberg, 2008). Before diagnosis, the absence of first words and sentences is the most frequently reported concern for parents (De Giacomo & Fombonne, 1998; Wetherby et al., 2004).

The most consistent language deficit in children with ASD is the pragmatic aspect such as the understanding of metaphors (Kalandadze, Norbury, Nærland, & Næss, 2016). Prosody and intonation patterns are also usually distinct from typically developing children (Tager‐Flusberg & Dominick, 2011). However, for the children that develop functional language, few articulatory problems are reported (Bartak, Rutter, & Cox, 1975). From a developmental perspective, the differences seen in children with ASD compared to typically developing children may be more quantitative than qualitative (Gernsbacher, Morson, & Grace, 2015; Gernsbacher, Morson, & Grace, 2016). Studies conducted by Norbury and colleagues lend support to the notion that the difference between children with ASD (with or without language impairments) and non‐ASD children (with or without language impairments) are dependent on the degree of language rather than the degree of autistic traits (see, for instance, Brock, Norbury, Einav, & Nation, 2008; Norbury, 2005).

#### 1.5.2 Syndromes with a known aetiology

*DS* or *Trisomy 21* is the most common known genetic cause of ID that is not inherited. Prevalence of DS has been reported in Europe and the US to be approximately 8 per 10,000 (Presson et al., 2013). For persons with DS, the gap between cognitive abilities and chronological age has been reported to increase into adulthood (Raitano Lee et al., 2016). A meta‐analysis indicated that individuals with DS show slow positive rates of change compared to what is expected in typically developing children (Patterson, Rapsey, & Glue, 2013). This development warrants the need for research focusing on effective best‐practice interventions. As delays and deficits in language are reported from early onset to adulthood, language interventions for this group are of particular importance (Martin, Klusek, Estigarribia, & Roberts, 2009).

Children with DS often score significantly lower than typically developing children on measures of expressive language (Finestack, Sterling, & Abbeduto, 2013; Næss, Lyster, Hulme, & Melby‐Lervåg, 2011). For receptive vocabulary, studies report mixed findings. Some studies indicate a clear challenge in expressive language relative to receptive language (e.g., Glenn & Cunningham, 2005; Laws & Bishop, 2003). Further, in a systematic review on language skills in children with DS, Næss et al. (2011) reported that receptive skills were not statistically significantly different compared to typically developing children with the same nonverbal mental age. However, other studies comparing children with DS to other mental age‐matched groups report difficulties in receptive language (Hick, Botting, & Conti‐Ramsden, 2005; Roberts et al., 2007). Additionally, deficits in syntax structure and complexity are quite common for the group (Martin et al., 2009). There are, however, large within‐syndrome variations (Abbeduto et al., 2016), and some of the differences and inconsistencies reported in the language domain may be due to variation in assessment procedures used in the studies, hearing loss, or variations in cognitive status across studies (Martin et al., 2009).

*Williams–Beuren syndrome*, also known as Williams syndrome, is a rare syndrome with prevalence reported to be approximately 1 in 7,500 (Strømme, Bjørnstad, & Ramstad, 2002). The syndrome is a multi‐system disorder caused by deletion of the Williams–Beuren syndrome chromosome region (Pober, 2010). Early onset developmental delays are typical for children with WS. However, clinical diagnostic criteria are typically not as useful for accurate diagnosis of WS compared to laboratory testing (Pober, 2010). For children with this syndrome, medical conditions apply to a much larger degree compared to that of typically developing children (Morris, Demsey, Leonard, Dilts, & Blackburn, 1988). The cognitive profile for this group are generally in the mild to moderate range for overall IQ, but there is variability within the range of approximate IQ scores between 40 and 100 (Martens, Wilson, & Reutens, 2008). The neurocognitive profile of WS is complex involving relative strengths in aspects of oral language and profound weaknesses in visuospatial cognition (Mervis and John, 2010).

It is perhaps due to the variations in the WS profile that has led some to conclude that language is within the normal range these individuals (Karmiloff‐Smith, 2007). Although studies indicate that some children with WS have strengths in expressive language, this strength is *relative to* other areas of functioning and not necessarily within the range found in typically developing children (Bellugi, Lichtenberger, Jones, Lai & St. George, 2000; Karmiloff‐Smith et al., 1997). Thus, there is a need for information considering language interventions for children with WS, especially considering that this has been an area with little focus since their language abilities may have been overstated in many ways (Brock, 2007; D'Souza & Karmiloff‐Smith, 2017).

*Fragile X syndrome* is the most common genetic cause of inherited ID. Prevalence estimates for FXS are approximately 1 in 5,500 for males (Macpherson & Murray, 2016) and approximately 1 in 8,000 for females. However, prevalence estimates vary considerably, especially due to advances in genetic testing (Hunter et al., 2014). Co‐occurrence with ASD is high in children with FXS, with up to 50% scoring above cut‐offs for an autism diagnosis on diagnostic tests for ASD (Hall, Lightbody, & Reiss, 2008). Early language milestones are delayed relative to typically developing children, and this difference is especially so for boys with FXS. The extent and nature of persistent language deficits are unclear due to mixed results from studies using different methodology and measures. One reason for imprecision in estimating language competence may be anxiety in the context of testing that these children can experience (Cornish, Sudhalter, & Turk, 2004). However, available evidence indicates impairments in language in children with FXS that includes both structural and pragmatic aspects of language, particularly vocabulary (Klusek, Martin, & Losh, 2014; Kover, McCary, Ingram, Hatton, & Roberts, 2015; Martin, Losh, Estigarribia, Sideris, & Roberts, 2013).

### 1.6 Overlap between the disorders

From a theoretical prospective, neuropsychology and neuroconstructivism give different explanations for neurodevelopmental disorders (D'Souza & Karmiloff‐Smith, 2017). On the one hand, neuropsychology points out that the brain has a modular structure characterised by distinct and highly specialised modules related to specific cognitive functions (see Obrzut & Hynd, 2013). The neuropsychological account suggests that genetic predispositions could cause a deficit in one or more innately specialised modules leading to different neurodevelopmental disorders (e.g., Frith, 1995; Leslie, 1992). On the other hand, neuroconstructivism suggests that children's brain presents specific neural patterns of activation but that the cognitive system is less specialised respect to adults (Johnson & de Haan, 2011; Johnson, 2011). Children's brain specialisation is actually supposed to increase over time as the results of the interaction between internal (i.e., psychological and neural subsystems) and external (i.e., environmental and social cues) factors (Mareschal et al., 2007). For this reason, impairments in one cognitive component could have effects on other cognitive system areas, constraining its development and higher‐level cognitive functions (Bishop, 1997; Karmiloff‐Smith, 1997). Although neuropsychology and neuroconstructivism differ in mechanisms involved in neurodevelopmental disorders, this complex debate points out the importance of cross‐syndrome comparisons to detect possible differences in children's neurodevelopmental disorders in terms of genetic, neural, cognitive, environmental features (D'Souza & Karmiloff‐Smith, 2017).

From a clinical perspective, children with a variety of neurodevelopmental disorders may present in a given context (e.g., special schools) and yet there is no single summary of the state of the art interventions that meaningfully impact child language outcomes for different neurodevelopmental disorders. In addition, clinicians will need to determine the most cost‐effective way of serving these different populations—are different treatment approaches warranted, or could children with different neurodevelopmental disorders but similar language learning needs benefit from a unified treatment approach? Such a comparison would elucidate whether similar treatment effect sizes obtain regardless of neurodevelopmental condition. Not only would such information be practically useful, but it would inform theories of atypical language development and commonalities in underlying mechanisms.

### 1.7 The intervention

The review addresses the effects of oral language interventions for children with neurodevelopmental disorders that are known to have atypical language development (i.e., the groups outlined in the previous section). We will include interventions that are delivered by clinicians and/or practitioners such as speech‐language pathologists, psychologists or teachers. Typically, these interventions will be delivered to the children in kindergarten, school, or in another clinical setting. We also include parent‐mediated interventions of language as these have gained interest in recent years (Abbeduto et al., 2016).

Notably, intervention approaches for improving skills in children with neurodevelopmental disorders derive from different theoretical frameworks. Broadly speaking, two main intervention approaches can be identified: (a) Applied Behaviour Analysis (ABA), and (b) interventions based on developmental psychological theory. While the former is founded on operant conditioning principles (Baer, Wolf, & Risley, 1968; Wolery, Bailey, & Sugai, 1988), the latter is based on interaction child‐oriented approach (see Sowden, Perkins, & Clegg, 2011). However, more eclectic approaches have been developed and have become more and more common (Schreibman et al., 2015). Although it is often difficult to classify interventions in a clear‐cut way, it is still possible to identify some components distinguishing the theoretical traditions. In the following sections some key aspects of ABA and the developmental psychological approach are described with one specific example of each approach.

In regard to the ABA approach, treatment protocols are characterised by operant conditioning, behavioural strategies (i.e., modelling, shaping, and chaining), highly structured settings, and a high number of hours for delivering the treatment (Baer et al., 1968; Lovaas, 1987). Among the ABA interventions, the “Discrete trial training” (DTT) aims to teach skills broken in discrete components and taught each of them one by one in subsequent steps (e.g., Smith, 2001). For example, the DTT training language is one‐to‐one intervention with child and adult working with table‐top exercises and where visual cues are presented to elicit verbal responses (Howlin, 1981). These activities are proposed for stimulating and improving expressive language, sentence formulation and verbal exchanges (Howlin, 1981; Krantz & McClannahan, 1981; Risley, Hart, & Doke, 1972).

As for the developmental psychology approach, interventions are characterised by a greater importance to interpersonal and interactive social exchanges, the presence of play activities and sharing child's activities, the promotion of affective engagement in child and adult relationship, and the vision of children as an active rather than a passive agent (Rogers & Lewis, 1989; Warren & Gazdag, 1990). An example of the developmental psychology approach intervention is the “Pivotal response training” (PRT) that is usually placed in a room where the child and the adult are asked to interact and play together (e.g., Pierce & Schreibman, 1995). The session is characterised by turn‐taken, frequent task variation, and the presence of natural stimuli (i.e., household object and toys) administrated in a flexible way (i.e., adapting to child spontaneous activities) to increase task motivation and better generalisation (Koegel & Koegel, 2006; Koegel, Koegel, Harrower, & Carter, 1999).

We aim to focus on interventions with a clear rationale indicating that the intervention content focuses on developing oral language based on methods descriptions in the publication. When such information is unclear or missing from descriptions in the included articles, we will search other publications or available documents online to determine whether language was a target of the intervention. Also, the outcome measures must be specific on language. This includes interventions specifically targeting the development of receptive and expressive vocabulary and semantics, grammar, narrative and other aspects of pragmatic language. Thus, we exclude social communication interventions (e.g., the PACT‐study (Green et al., 2010) that focus more on precursor skills, such as joint attention, and where changes in autism symptoms is the primary outcome measure, from the review although such studies often also measure change in oral language as a secondary outcome measure.

The control condition should be a passive control group, active control group or waiting list control group. Studies with no control group will be excluded from the review.

#### 1.7.1 Examples of studies to be included in the review

One example of a study that is eligible for inclusion in the review is Burgoyne and colleagues’ reading and language intervention for children with DS (Burgoyne et al., 2012). The study was a randomised controlled trial (RCT) design and involved 57 children with DS enroled in mainstream primary schools in the UK. The language intervention was delivered by trained teaching assistants that worked individually with the child for daily 40‐min sessions over a period of 40 weeks for the intervention group. The waitlist control group received 20 weeks of treatment as usual before receiving the same intervention for the last 20 weeks. Assessments were conducted at baseline, after 20 weeks of intervention and after 40 weeks of intervention. Effect sizes were reported favouring the intervention group on measures of taught expressive vocabulary (*d* = 0.47 *p* = 0.011) and single word reading (*d* = 0.23 *p* = 0.002) after 20 weeks of intervention. The difference between the original intervention group and the waitlist control after the former had received 40 weeks of intervention, whereas the latter had received 20 weeks, were taught expressive vocabulary; *d* = 0.42, *p* = 0.064 and single word reading *d* = 0.22, *p* = 0.055, but no transfer effects were found indicating little generalisation of skills to other domains not taught in the intervention.

Another eligible study for inclusion is the RCT that was conducted by Buschmann and colleagues (2009), focusing on children with very specific deficits in expressive language aged 24.7–27 months. This study was a parent‐based language intervention lasting three months with seven sessions of 2 hours and a 1‐hour session 6 months later. The intervention was a highly structured interactive group‐based programme (5–10 participants in each group). Picture book sharing was one of the main topics in the intervention building on the rationale that child‐oriented interactions and parents as models may enhance children's language abilities. Final analyses were conducted on a sample consisting of *n* = 24 in the intervention group and *n* = 23 in the waitlist group. The study also included a comparison language‐normal group consisting of *n* = 36. Effect size estimates at follow‐up ranged from *d* = 0.23 on plural forming to *d* = 1.16 on syntax measured by parent report, all in favour of the intervention group compared to the waitlist condition (Buschmann et al., 2009).

In the next section, we describe detailed aspects of how intervention might work related to specific elements of the target interventions in this review.

### 1.8 How the intervention might work

Whether an intervention is effective or not relies on several variables. Intervention content is critical but other variables are also important, such as who delivers the intervention and in what context (home, school or clinics), can also influence the results. Further, the dosage, or the frequency, intensity and duration of the intervention, may influence the outcome (Storkel et al., 2019; Justice, Chen, Tambyraja, & Logan, 2018) and may also be important factors driving decisions related to more practical and political aspects of service delivery, such availability of staff and financial costs of the intervention. Below are short descriptions of some key factors that will be closely monitored in the planned systematic review.

#### 1.8.1 The delivery agent

An important aspect of intervention research relates to who delivers the intervention. Evaluations of efficacy versus effectiveness of interventions where the former typically involves expert clinicians at university clinics, and effectiveness interventions mainly involve delivery of interventions in the child's preschool or school delivered by the staff that work with the child on a day to day basis, such as teachers, or by the parents of the child at home. Although efficacy trials are important, it is also crucial for broad implementation at the community level so that interventions that proves to be effective can be delivered in ways that are manageable both in terms of cost and time efficiency. Following the dichotomisation of efficacy and effectiveness, the strength of the former is that it is easier to control extraneous variables that increase internal validity. This control is an important feature when wanting to infer causation. However, the increased internal validity comes at the cost of external validity and generalisation. To generalise and determine whether interventions can be implemented in everyday contexts by non‐specialists, effectiveness studies are also very important. Thus, this review includes efficacy *and* effectiveness trials that are parent‐implemented or delivered by persons working with the child at preschools/schools or in other more clinical settings.

#### 1.8.2 The context of delivery

As the present review includes children with neurodevelopmental disorders, the context of delivery is especially important considering the challenges many of these children may display in transferring skills taught during the intervention to other contexts. The context of delivery will typically be in preschools and kindergartens, in schools, in clinical settings (including University labs), or in the child's home. Within these settings the context may be for instance one‐to‐one adult‐child interaction or in groups with other children and an adult. The contexts will also differ as to how structured the setting might be. Some interventions can be highly stringent table‐top training with a strict intervention‐manual (e.g. ABA), whereas other interventions can be floor‐based play session with less stringency (typically developmental approaches). The delivery agents will vary depending on the context in the various studies included but typically parents will be the delivery agents when the intervention is delivered in the home, preschool–kindergarten‐ and school‐teachers, and assistants in preschool and schools, and clinical staff and University psychologists in clinical and University lab settings. However, clinical staff and speech‐language pathologists might also be frequently used in interventions in preschool and school settings as well.

#### 1.8.3 The dosage

The amount of intervention required to affect change is a topic of heated debate; it is therefore noteworthy that very little systematic research has investigated the extent to which outcomes depend on intervention frequency, duration or intensity (Warren, Fey, & Yoder, 2007). Dosage may refer to the total number of therapy hours a child completes, but may also include other methods of delivery such as booster sessions to revive or sustain an intervention effect following the initial intervention period. Unfortunately, dosage is an important aspect of intervention research as it is inevitably tied to time‐, resource‐ and cost‐efficiency constraints. It might be that some neurodevelopmental disorders require differing dosages to achieve the same treatment effect. Such information can be critical when planning effective services.

#### 1.8.4 The outcome measures

**Figure 1:**
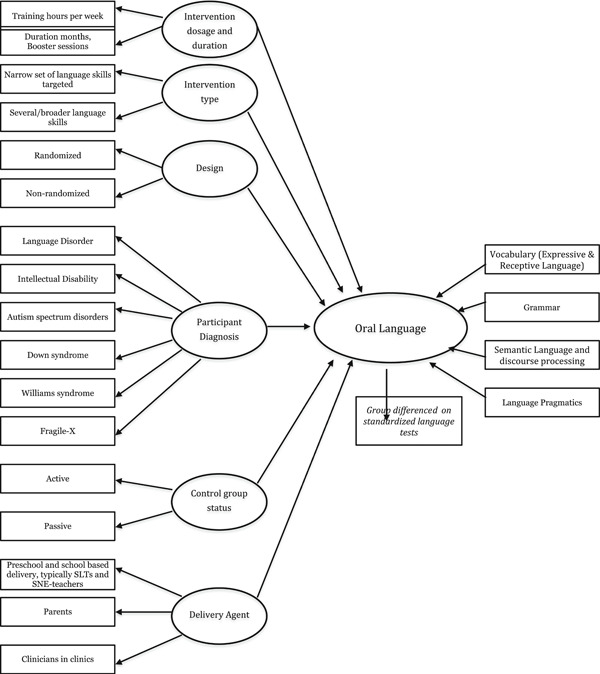
Theoretical model of how the different variables may relate to oral language

Measures and measurement techniques have different strengths and weaknesses. Among other things, measures can be based on direct observation or informant report; data can be derived from a standardised assessment protocol belonging to a particular intervention type or be based upon free‐play, or measures may be rated by blinded coders or designed to be responsive to change over time. Further, intervention studies using outcome measures more proximal to intervention targets, compared to more distal measures, typically report larger treatment effects (Green et al., 2010; Nordahl‐Hansen, Fletcher‐Watson, McConachie, & Kaale, 2016; Yoder, Bottema‐Beutel, Woynaroski, Chandrasekhar, & Sandbank, 2013). The specific measures of outcome in this systematic review are listed in the theoretical model below (see Figure 1).

#### 1.8.5 The child's cognitive status

Historically, diagnostic criteria for neurodevelopmental disorders have employed inclusion and exclusion criteria that relate to whether non‐verbal IQ is over or below certain threshold levels. For instance, to be diagnosed with LD, nonverbal IQ had to be within the “normal range” and sometimes discrepancies between verbal and nonverbal abilities were required However, the trend in the DSM‐5 is to downplay the role of cognitive levels as measured by traditional intelligence tests and to focus more on adaptive functioning. Similarly, the CATALISE consortium clearly rejected the use of non‐verbal ability as an exclusion criteria for LD (Bishop et al., 2016) and does not appear to associate with rate of language change, at least in the primary school years (Norbury et al., 2017). Research evidence regarding the role of nonverbal cognitive ability in response to treatment is lacking and urgently needed. Cognitive functioning remains closely intertwined with neurodevelopmental disorders and poses a key variable that may influence intervention outcomes (Bishop & Edmundson, 1987; Rice, 2016).

#### 1.8.6 Commonalities and differences across neurodevelopmental disorders

The selected neurodevelopmental disorders included in the present systematic review have many similarities in oral language profiles. These similarities mean that effective interventions for children with one type of neurodevelopmental disorder may also be effective for children with other neurodevelopmental conditions. However, there are also unique cognitive and behavioural profiles that may influence both the natural course of language development and the response to interventions. Including a range of neurodevelopmental‐disorders will allow for an overall impression of the impact of oral language interventions, as well as comparative analyses of effect sizes across neurodevelopmental conditions.

Figure 1 below depicts a theoretical model of how the interventions might work.

### 1.9 Why it is important to do the review

There is a need for mapping of interventions across neurodevelopmental conditions to gain better understanding of underlying mechanisms of atypical language development (Abbeduto et al., 2016). The lack of pairwise comparison of neurodevelopmental disorders is a gap in the research literature since much research has focused on narrow criteria of inclusion, focusing on “pure” groups that mirror real world clinical contexts to a lesser degree (Bishop et al., 2016; Rice et al., 2005). Further, the need to investigate variation in treatment effects following interventions for the neurodevelopmental disorder groups included in this review in relation to nonverbal IQ is highly warranted since evidence that such variables influence outcomes is scarce (Norbury et al., 2016). This issue is particularly relevant considering changes in diagnostic criteria and an inability to generalise previous intervention studies of children with LD to other clinical groups because previous intervention studies of children with LDs often excluded children with nonverbal IQ below 85. Finally, the review is also important because it vill give an overview of the empirical coverage and also on what area there is need for new studies and replication studies.

Meta‐analyses evaluating effects of language interventions have focused on children with what may be termed “specific” LDs (Cirrin & Gillam, 2008) or primary speech and/or LDs (Law, Dennis, & Charlton, 2017). Many of these meta‐analyses exclude neurodevelopmental disorders such as ID, DS, ASD, WS, and FXS. An exception is the meta‐analysis conducted by Roberts and Kaiser (2011), where children with “all types of language impairments” in addition to intellectual impairments and ASD were included. Children with language impairments of both known and unknown origin were included, as well as children with and without intellectual disabilities. As such, the Roberts and Kaiser study included multiple disorders within their meta‐analysis without comparing effects between the disordered groups. However, the present protocol goes further in that we also categorise the disorders in terms of diagnostic status, and may thus provide additional knowledge for the particular disorders under scrutiny. Furthermore, the Roberts and Kaiser review included only parent‐implemented interventions, whereas our proposed review considers clinician and educator led interventions which may be particularly relevant to older children.

Table 1 lists the reviews that are most closely related to the review we aim to do. However, as apparent from the list, there are no reviews that focus on broad inclusion of diagnostic groups in a cross‐disorder manner. With respect to the ongoing Cochrane review by Law and colleagues (2017), while Law et al. include child LDs, their review is limited to children without co‐occurring developmental conditions. This review will therefore overlap with the present proposal, but our review will include children with additional developmental disorders and more inclusive non‐verbal cognitive abilities. Our proposal uniquely considers the success of interventions for oral language across a broader range of populations and contexts, providing more ecological validity to our findings.

**Table 1:** Examples of systematic reviews of language interventions for the target groups of the present review

Authors, year	Diagnosis/inclusion criteria	Main findings	Design of the studies included	Meta‐analytic method	Participant characteristics	Intervention characteristics	Moderators
Law, Garrett, and Nye, (2004)	Primary speech and language delay	Expressive vocabulary d = 0.98 (k = 2) Expressive syntax d = 0.70 (k = 5) Receptive syntax d = −0.04 (k = 2)	RCTs	Random‐effects models were used, not clear if d was corrected for small sample size	Participants were children or adolescents with primary speech and language difficulties	Interventions that aimed to improve expressive or receptive phonology, syntax or vocabulary were examined. Interventions were implemented by parents or clinicians	Secondary analyses: removing 1) Parent‐administered interventions, 2) duration of less than 8 weeks, 3) removing studies with severe receptive language difficulties.
Cirrin and Gillam (2008)	Language disorder (spoken language disorders)	Syntax and morphology d = −.03 to 1.3 (k = 3) Semantics and vocabulary d = .5–3.5 (k = 4) Phonological awareness and metalinguistics d = .29–1.78 (k = 3) Language processing d = .09–1.34 (k = 2)	Experimental and quasi‐experimental studies using a control group. Multiple‐baseline single‐case design	Effect sizes corrected for pretest differences when pretest data were available. Percentage of nonoverlapping data used in N=1 design	Age 4–14	Broad (delivery, dosage, context) specific language interventions. Efficacy and effectiveness studies	No quantitative moderator analysis
Cirrin et al. (2010)	Speech/Language impairments as either primary or secondary disability	Vocabulary *d* = −0.10to 1.65 Language and Literacy *d* = −.39 to 0.15	RCTs, Systematic reviews of RCTs, nonrandomized comparison studies, multiple‐baseline single‐case designs	Effect size *d* for RCTs and percentage of nonoverlapping data for single‐case designs	Age 5–11	Different speech‐language intervention service delivery models	No quantitative moderator analysis
Roberts and Kaiser (2011)	Any type of primary *and* secondary language impairment including developmental disorders but not specified (1 SD below mean on standardised language assessment or <50 expressive words at age 2)	When considering only custom measures, the effect size was correlated with control group strength and experiment vs quasi‐experimental design. High levels of discussion were associated with larger effect sizes	Experimental and quasi‐experimental studies using a control group	Random‐effects model for effect sizes. Hedges *g* used to adjust for small sample sizes	Age 18–60 months	Studied interventions with the goal of increasing word knowledge or comprehension that could be implemented in a classroom setting	Disability type, Measure type
Gerber, Brice, Capone, Fujiki, and Timler (2012)	Language impairments w. strict exclusion of children with social communication impairments such as ASD, DS, ID, etc.	Need for future studies	Case studies, case series, single‐subject design, pretest‐posttest group design	7 of 8 studies descriptive. Cumulative effect sizes not possible	Age 5–11 years	Social language interventions	N/A
Law et al. (2017)	Primary speech and/or language disorder (excludes children with learning disabilities)	N/A (Review under construction)	RCTs	Plans to use Effect size and Odds Ratio with Confidence intervals	Up to 18 years	Interventions that aim to improve expressive or receptive phonology, syntax or vocabulary will be examined. Interventions implemented by any	N/A

Our results will also elucidate whether there are differences in response to intervention between disorders, which can enhance our understanding of whether tailored treatment plans are needed for the specific disorders. Thus, the present review will be of high clinical importance and may guide clinicians, therapists, practitioners and parents in selecting optimal interventions for these children.

From a societal perspective, this systematic review can influence the development of policy and best practice for children with neurodevelopmental disorders. In addition to covering various disorders, we also use a broad age range of inclusion from preschool years to school age years in order to map not only the effect of early interventions, but also the potential for language change in older children. A heightened focus on oral language interventions for school‐aged children is needed as despite a focus on early intervention, LDs are often persistent and the language needs of educational curricula and social interactions increases in complexity over time (Norbury, 2015). This focus also taps into a topic of debate within the speech‐language therapy community regarding the optimal age when children may be most responsive to intervention. Thus, we will also look at timing of intervention comparing early preschool to secondary school interventions.

It may be worth emphasising that interventions targeting language in children are plagued by lack of rigour, especially considering provisions of a sound theoretical rationale and evidence for efficacy (Hulme & Melby‐Lervåg, 2015). Contributions to build a sounder evidence base in this field are therefore critical and can give information about what works as well as uncover what does not. The proposed review will also highlight areas where evidence is lacking and provide an overview of evidence quality for a range of neurodevelopmental disorders.

## 2 OBJECTIVES

The primary objective for this review is to evaluate the effect of interventions that aim to increase language skills in children with different neurodevelopmental disorders. Another primary aim is to identify interventions that have similar impacts (effect size differences) across these different disorders. Thee groups of children included in the review have the following diagnoses: ASD, ID, DS, Fragile X, LD, and WS. This review will map the kinds of oral language interventions that are available for the respective disorders and can as such be used as a synthesis for researchers, clinicians, policy‐makers and other stakeholders.

The main research questions addressed in this review are:

- How effective are oral language interventions for children across different neurodevelopmental disorders?
- Do the effects of the oral language interventions differ between groups of children with different neurodevelopmental disorders?
- Are treatment effects moderated by nonverbal intelligence?
- What aspects of language appear more malleable to intervention?
- What additional factors influence response to treatment? The factors tested will include dosage (frequency, intensity and duration), delivery agent (parent‐mediated, clinician, school staff, research team), child age, and where possible, treatment focus (e.g., general language stimulation, shared book reading, parent/teacher training).

## 3 METHODOLOGY

### 3.1 Criteria for including and excluding studies

#### 3.1.1 Types of study designs

- We will include quantitative studies that use a randomised experimental or a quasi‐experimental design with a control group.
- The studies have to include baseline measures to make it possible to evaluate whether groups are sufficiently equivalent and comparable prior to intervention onset.
- Studies must report posttest measures to enable computation of group differences and change following the interventions.
- Quasi‐experimental designs with control groups are included in the review as it would otherwise be difficult to obtain a large enough pool of studies from which to derive recommendations.

#### 3.1.2 Types of participants

- We will include studies of children with neurodevelopmental disorders that are are characterised by oral language deficits. This list includes children with ASD, ID, DS, Fragile X, LD, and WS. To be as inclusive as possible, we do not impose a priori cut‐offs for level or profile of language deficit required for inclusion in this review.
- Inclusion criteria for age range will be 2 to 18 years, comprising the preschool and school years for typically developing children.
- Excluded: Studies of children described as having primary speech sound disorders, such as those related to oral‐motor function, articulation, and dyspraxia, where the primary intervention target in improving speech intelligibility (Cohen, 2001).

#### 3.1.3 Types of interventions

There is an approach to intervention that focuses on general cognitive training (such as working memory training, training of executive functions or auditory processing) for children with neurodevelopmental disorders. Earlier systematic reviews indicate that intervention effects tend to be limited to similar training tasks and do not transfer to specific oral language targets (Melby‐Lervåg & Hulme, 2013; Strong, Torgerson, Torgerson, & Hulme, 2011). We exclude these these interventions and focus this review on targeted oral language interventions that include language‐based tasks and specific language targets as outcome measures.

- Intervention studies employ a variety of theory driven and behavioural techniques to improve oral language skills. These may include general language stimulation, shared book reading, explicit instruction of vocabulary, narrative structure or grammatical rules, milieu teaching, training to enhance parent language and communication input, etc. We recognise that some studies will use eclectic approaches or may not specify a particular approach. We also note that there may be too few instances of individual approaches to be able to determine if one approach is more effective in some neurodevelopmental conditions relative to others. We will make every effort to code the intervention approach employed.
- The types of oral language targets that will be included in the review are standardised tests of receptive, expressive and total language, standardised and bespoke measures of vocabulary, grammar, narrative, discourse processing and pragmatic language, in both receptive and expressive modalities.
- As the focus of this review is on interventions of language and not on speech‐interventions we exclude interventions that focus on phonological skills and/or articulation skills and studies with interventions that solely target the phonological domain such as oral‐motor musculature interventions related to speech impairments. However, some interventions will not make clear‐cut distinctions between speech and language. These studies will be included after evaluation if (a) the study or available information of the intervention clearly states that oral language (as described above) is part of the intervention content, and (b) if the outcome measures match the above noted specific targets of language.

#### 3.1.4 Types of outcome measures

The planned primary outcome measures that will be included in this systematic review are the ones that target oral language broadly defined (see Figure 1). Some examples of assessment tools targeting oral language include:

- Expressive and receptive vocabulary (e.g., *Expressive Vocabulary Test* EVT‐2; Williams, 2007, *British Picture Vocabulary Scale* BPVS; Dunn, 2009)
- Expressive and receptive grammar and syntax (e.g., Test for reception of Grammar‐2; Bishop, 2005; Renfrew Action Picture Test)
- Narrative comprehension and retelling (e.g., *Test of Narrative Language*; Gillam & Pearson, 2004; ERNNI, Bishop, 2003)
- Pragmatic use of language in communication (e.g., Test of Pragmatic Language, TOPL‐2; Phelps‐Terasaki & Phelps‐Gunn, 2004). However, note that for this outcome we will include only inferencing, figurative language use and discourse skills (i.e., measures that directly taps oral language skills)
- Omnibus tests of language, such as the *Clinical Evaluation of Language Fundamentals* (CELF‐4UK; Semel, Wiig, & Secord, 2006) and *Test for Auditory Comprehension of Language* (TACL; Carrow‐Woolfolk, 1985)
- We will not include measures of social communicative skills (such as eye contact, conversational repair, topic maintenance) as outcome measures for this systematic review.

We will mainly focus on tests that assess oral language skills in children directly. We are including both standardised tests and custom made bespoke test materials. However, if direct tests are not available, we will also include parental, clinician or teacher reports of language (such as the M‐CDI) as well as curriculum‐based measures (e.g. speaking and listening attainment scores. Assessment method can also potentially be an important moderator variable.

#### 3.1.5 Types of settings

We will include studies that report on interventions that are directly delivered to the child, individually or in groups, from another person or persons. The setting of delivery will be in:

- Preschools and kindergartens
- In schools (typically by education staff such as teachers or learning support assistants)
- Clinical setting (typically by clinical staff such as speech‐language therapists)
- In the child's home for parent‐mediated interventions

#### 3.1.6 Delivery agents

For this systematic review, we plan to include the following agents, who will be delivering the interventions:

- Special education teachers
- Clinical staff such as speech‐language therapists and psychologists
- Teachers
- Assistants
- Parents

We exclude the following interventions on the grounds that they are not the main and traditional delivery agents of oral language interventions for the diagnostic groups included in this review. Dietary and pharmaceutical interventions are typically more related to the field of medicine and do not target language specifically. Animal‐assisted interventions do not target the enhancement of language but focus on adaptive communication. Computer‐assisted interventions typically include very brief manipulations in experimental lab‐settings and fall outside of the traditional delivery agents targeted in this review.

- Report on dietary interventions
- Report on pharmaceutical treatments
- Report on non‐person delivered interventions such as through computers or animal‐assisted interventions

#### 3.1.7 Duration of follow‐up

We will collect data from immediately after post‐treatment but also from long term follow‐up where available.

#### 3.1.8 Search strategy

Due to risk of language bias, no restrictions on language will be included in the search. We will seek translations if necessary. Studies included will be for the time‐period from 1946 to the present which is the span covered by, for example, MEDLINE. We will use multiple sources for information retrieval. We will consult with expertise from the Norwegian Cochrane and Campbell offices for the electronic searches and the search in other resources as well as it will be supervised by a specialist in information retrieval at the Library of Humanities and Social Sciences, University of Oslo. We will use Endnote as well as Distiller for storage of citations.

Details of the search strategy are included in Appendix A.

#### 3.1.9 Electronic searches

We plan to search the following databases:

1. The Cochrane Library
2. The Campbell Library
3. MEDLINE
4. EMBASE
5. CINAHL EBSCO
6. Academic Search Complete (EBSCO)
7. LILACS (Latin American and Carribbean Health Sciences Literature)
8. SpeechBITE
9. PsycINFO
10. ERIC
11. Education Source (EBSCO)
12. British Education Index (EBSCO)
13. Epistemonikos
14. ClinicalTrials.gov
15. Web of Science
16. ProQuest Digital Dissertations
17. Linguistics and Language Behavior Abstracts (LLBA)
18. Scopus Science Direct
19. Google Scholar

A list of search terms that will be used to identify articles is presented in Appendix A.

#### 3.1.10 Search in other resources

1) Scanning reference lists in meta‐analyses (see Table 1, Appendix I).
2) The listserv of the Society for Research on Educational Effectiveness and Society for the Scientific Study of Reading will be used to ask researchers for in‐press or unpublished material.
3) A manual review of the tables of contents of the following key journals will be conducted: Journal of Child Psychology and Psychiatry, Journal of Autism and Developmental Disorders, International Journal of Language and Communication Disorders, Journal of Intellectual Disability Research.

5) Unpublished reports, such as dissertations, technical reports, and conference presentations, will be located via searches in

– OpenGrey.eu
– Proquest Dissertations and Theses
– PDF search in Google. The advanced search option will be used in the Google search. Additionally, the words “study”, “studies” and “control group” will be used to further limit the search as per the advice given in the Campbell systematic review information retrieval guide (Kugley et al., 2016).

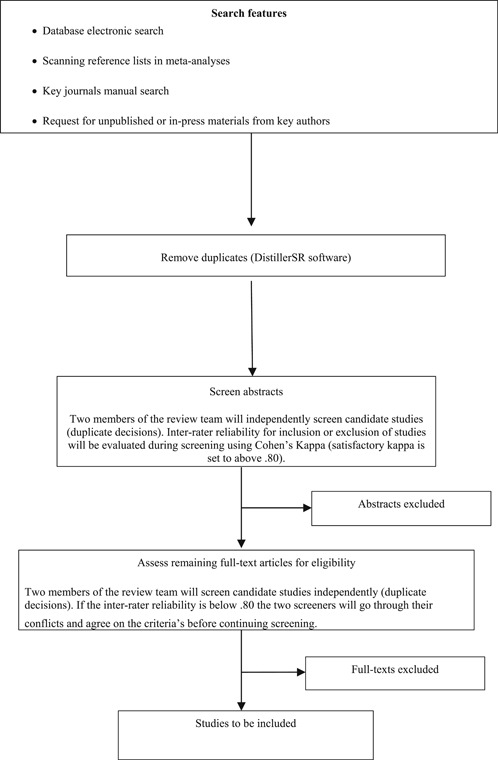

### 3.2 Description of methods used in primary research

Although there will be some studies using randomised controlled trials, we expect the largest proportion of intervention studies to have employed a quasi‐experimental design with a control group, which is the reason for including the latter type of study designs in the systematic review.

### 3.3 Criteria for determination of independent findings

Due to the possibility of obtaining biased estimates if overall effect sizes from one study are computed more than once, some prior considerations before study coding will be made.

#### 3.3.1 Multiple reports of the same study

There may be several reports for one study. The different reports may contain additional information. We will extract the most useful and important information needed for each item in the coding manual. As multiple reports of the same study may lead to incorrect weighting of study results, we will contact authors and investigators when we are uncertain about multiple publication of original research.

#### 3.3.2 Multiple studies in single reports

If more than one study is described in a single report, each study within the report will be coded separately.

#### 3.3.3 Multiple comparison groups and multiple interventions

Some studies may have used more than one control group. In our analyses, we will include only the neurodevelopmental disorder groups that meet our eligibility criteria. Some studies may compare the same control groups to different treatment groups, and these groups may be included in the same analysis of mean effect size for treated and untreated controls. We will include these studies in the analysis but assume zero correlation between the outcomes.

#### 3.3.4 Multiple outcomes

If studies use more than one indication for the same construct, we will use the mean of the indicators when possible. We will document to what extent studies report a priori primary and secondary outcome measures.

#### 3.3.5 Multiple time points

We will use pretest, immediate posttest and any follow‐up measures regardless of timeframe.

### 3.4 Details of study coding categories

Since systematic differences between studies may influence the outcome effects we will categorise and code variables related to the following:

- Disorder and diagnostic status
- Year study is published and type of publication
- Sample characteristics including age/grade level, language status and developmental level/IQ
- Study quality (e.g., design; recruitment; sample size; type of control group; attrition)
- Intervention/implementation characteristics (setting, mode of delivery; instructor; group size; dosage of intervention, type of intervention)
- Type of language difficulty targeted
- Session duration
- Outcome (name of test; type of test; global vs. specific measure)
- Effect size coding

#### 3.4.1 Procedures for making inclusion/exclusion decisions

Two coders will independently screen titles, abstracts and full‐texts. Kappa statistics will be reported to indicate level of agreement. Two of the authors will do the study coding independently, and we will duplicate dual data extraction to reduce risk of making mistakes and a single person's bias. If encountering missing or unclear information on key variables, we will contact the authors responsible in order to obtain coding information and remove ambiguity. If key information is still unavailable, the variable will be coded as missing. When coders disagree on inclusion and exclusion the particular studies will be discussed in relation to the criteria set up for including and excluding studies. If agreement is not reached the last author will be consulted.

#### 3.4.2 Examining the strength of evidence

We will adapt the Grading of Recommendations, Assessment, Development and Evaluation system (GRADE; Guyatt, Oxman et al. 2008; 2011) to assess the body of evidence. We plan to assess the overall quality of outcomes as high, moderate, low or very low. The intervention studies are rated based on the limitations of the study, the inconsistency or heterogeneity of the results, the indirectness of the evidence, as well as imprecision and reporting bias.

### 3.5 Statistical procedures and conventions

The “Comprehensive meta‐analysis” programme (Borenstein, Hedges, Higgins, & Rothstein, 2005) will be the main platform for conducting the statistical analyses. When sample sizes are small we will analyse effects using Hedges *g (*Hedges & Olkin, 1985). This method allows to compare baselines between intervention‐ and control –group in quasi‐experimental designs. As recommended by Morris (2008), effect sizes will be calculated by subtracting the posttest mean from the pretest mean in each group, and then by subtracting the gain in the control group from the gain in the intervention group. The result will be then divided on the pooled standard deviation. The effect size will be also corrected for a pre posttest correlation of 0.5 that could be a reasonable estimation of pre post correlation in these kind of studies. Effect sizes for follow‐up tests will be calculated using data from pretest and final time of follow‐up.

Analyses will depend on the number of studies obtained from the searches.

When analysing mean effect sizes, we will use a random‐effects model calculating weighted average of individual study effects. The choice of random‐effects model is because it is highly unlikely to assume a common effect size for the studies that will be included in this systematic review (Borenstein & Higgins, 2013).

In addition to calculating the mean effect size, it is important to address the variability between results and how the various studies are dispersed about the mean (Borenstein, Higgins, Hedges, & Rothstein, 2017). To identify and measure the heterogeneity among studies, we will use a set of statistics. We will use the *Q*‐statistic that provides a test of the null hypothesis that all studies in the analysis share a common effect size. We will use the *I* ^2^ statistic to get indications of whether the observed variance reflects differences in true effect sizes rather than sampling error. We will report the *T* ^2^ statistic that are the variance of true effect sizes obtained from the various studies. We will also report *T*, that is, the standard deviation of true effects. We will also compute the 95% prediction interval (mean ±2T).

Moderator analyses may elucidate important differences. Figure 1 shows the model for the review. Preferably, we would have liked to test the whole model using meta structural equation modelling. However, the expected number of studies and studies that report correlations in this area is unlikely to be sufficient to do meta‐SEM. We will therefore use

meta‐regression procedures to test aspects of the models in different analyses. Rather than using MASEM, it is likely that we will use method of moments meta‐regression for continuous variables (e.g., age, duration of intervention, etc.). To examine whether effects on language comprehension are mediated through language comprehension gains, we will set up mediation models using meta‐regression. For categorical moderator variables, studies will be separated into subsets based on the categories in the moderator variables, for instance experiments versus quasi‐experiments.

To examine differences in effect sizes between subsets in the study‐sample, we will use a *Q*‐test. However, due to expected heterogeneity across studies, when final searches do not include more than five studies in a subset (*k* < 5), this analysis will not be conducted. The overlap between confidence intervals will be used to examine the size of the difference between subsets of studies.

We will make efforts to retrieve studies from the grey literature to use as moderator when possible, in line with recommendations for meta‐analysis conduct (Higgins & Green, 2011).

We plan to test only the moderators for which there are clear theoretical motivations for testing as increasing the number of moderators can result in type 2 errors.

Special care will be taken regarding publication biases. Publication bias refers to the notion that a mean effect size can be upwardly biased because only studies with large or significant effects get published (i.e., file‐drawer problem with entire studies), or that authors report only data on variables that show effects (Simmons, Nelson, & Simonsohn, 2011).

To estimate the impact from publication bias statistically, a common technique is to use funnel plots in combination with a trim‐and‐fill analysis. However, this method can be flawed (Lau, Ioannidis, Terrin, Schmid & Olkin, 2006). Instead, we will use the *p*‐curve method that surpass some of central weaknesses in the funnel plot/trim‐ and‐fill analysis (Simonsohn, Nelson, & Simmons, 2014). A *p*‐curve contains plots from the distribution of *p‐*values (*p* < .05) in published studies. The shape of the *p*‐curve is a function of the effect size and sample size when the power level is taken into account. If there are true effects, the distribution of published *p*‐values should be right‐skewed with more low (.01 s) than high (.04 s) *p‐*values. On the other hand, in studies that are affected by publication bias (because researchers discard entire studies or discard analyses or parts of studies), the *p*‐curves are left‐skewed or flat and provide no support for an effect size of considerable magnitude (“no evidential value”).

We expect instances of missing data. If data are critical to calculate an effect size, articles with missing data will be excluded if authors of the study do not respond to requests to provide these additional data. In cases where an effect size can be computed but on no other outcomes or moderator variables, the study will be included in all the analyses for which sufficient data were provided.

### 3.6 Treatment of qualitative research

We do not plan to include qualitative research.

## APPENDIX A EXAMPLE PSYCINFO SEARCH TERM STRATEGY

Search filters:

Participant age: up to 11 years 11 months

1. Intellectual disabilit*
2. Intellectual disorder*
3. Developmental disorder*
4. Developmental delay*
5. Developmental disabilit*
6. Mental ADJ2 retard*
7. Language Disorder
8. Specific Language Impair*
9. SLI
10. Developmental Language Disorder
11. Language Delay*
12. Language defic*
13. Language impair*
14. Language problem*
15. Semantic‐pragmatic disorder*
16. Expressive language disorder*
17. Pervasive development* disorder*
18. PDD
19. PDD‐NOS
20. Autis*
21. Asperger
22. ASD
23. Down Syndrome
24. Trisomy 21
25. Chromosome ADJ1 21
26. Down* Disease
27. (mongol OR mongols OR mongoloid OR mongolism OR mongolianism)
28. Fragile X
29. FXS
30. Fragile ADJ1 X
31. Chromosome ADJ1 X
32. Marker X syndrome
33. Martin‐Bell syndrome
34. Williams Syndrome
35. Williams Beuren Syndrome
36. WS
37. OR/1–35
38. language ADJ3 intervent*
39. Language ADJ3 treat*
40. Language ADJ3 educat*
41. language ADJ3 therap*
42. language ADJ3 train*
43. language ADJ3 facilit*
44. language ADJ3 program*
45. OR/37–42
46. 36 AND 43
47. Vocabulary
48. Word ADJ3 knowledge
49. Word ADJ3 learn*
50. Linguistic ADJ3 comprehen*
51. Language ADJ3 comprehen*
52. Listening ADJ3 comprehen*
53. Language proficien*
54. Language skill*
55. Language abilit*
56. Language acquisition
57. Oral ADJ2 language
58. Spoken ADJ2 language
59. Gramma*
60. Syntax
61. Syntactic*
62. Semantic*
63. Morph*
64. Narrative skill*
65. Narrative comprehen*
66. Figurative ADJ3 speech
67. Figurative ADJ3 language
68. Figurative ADJ3 pragmatic*
69. OR/44–62
70. experiment*
71. randomi#ed controlled trial
72. controlled clinical trial
73. randomi#ed
74. randomly
75. trial
76. group*
77. quasi ADJ1 experiment
78. or/64–71
79. 36 AND 43 AND 73
